# A comparative impact evaluation of two human resource models for community-based active tuberculosis case finding in Ho Chi Minh City, Viet Nam

**DOI:** 10.1186/s12889-020-09042-4

**Published:** 2020-06-15

**Authors:** Luan Nguyen Quang Vo, Rachel Jeanette Forse, Andrew James Codlin, Thanh Nguyen Vu, Giang Truong Le, Giang Chau Do, Vinh Van Truong, Ha Minh Dang, Lan Huu Nguyen, Hoa Binh Nguyen, Nhung Viet Nguyen, Jens Levy, Bertie Squire, Knut Lonnroth, Maxine Caws

**Affiliations:** 1 Friends for International TB Relief, 68B Nguyen Van Troi, 8, Phu Nhuan, Ho Chi Minh City, Viet Nam; 2 Interactive Research and Development, Ho Chi Minh City, Viet Nam; 3 Ho Chi Minh City Public Health Association, Ho Chi Minh City, Viet Nam; 4 Pham Ngoc Thach Hospital, Ho Chi Minh City, Viet Nam; 5 National Lung Hospital, Ha Noi, Viet Nam; 6 KNCV Tuberculosefonds, The Hague, The Netherlands; 7 Liverpool School of Tropical Medicine, Department of Clinical Sciences, Liverpool, UK; 8 Karolinska Institutet, Department of Global Public Health, Stockholm, Sweden; 9 Birat Nepal Medical Trust, Lazimpat, Kathmandu, Nepal

**Keywords:** Comparative impact evaluation, Human resource model, Employees, Volunteers, Community health workers, Tuberculosis, Active case finding, Viet Nam

## Abstract

**Background:**

To achieve the WHO End TB Strategy targets, it is necessary to detect and treat more people with active TB early. Scale–up of active case finding (ACF) may be one strategy to achieve that goal. Given human resource constraints in the health systems of most high TB burden countries, volunteer community health workers (CHW) have been widely used to economically scale up TB ACF. However, more evidence is needed on the most cost-effective compensation models for these CHWs and their potential impact on case finding to inform optimal scale-up policies.

**Methods:**

We conducted a two-year, controlled intervention study in 12 districts of Ho Chi Minh City, Viet Nam. We engaged CHWs as salaried employees (3 districts) or incentivized volunteers (3 districts) to conduct ACF among contacts of people with TB and urban priority groups. Eligible persons were asked to attend health services for radiographic screening and rapid molecular diagnosis or smear microscopy. Individuals diagnosed with TB were linked to appropriate care. Six districts providing routine NTP care served as control area. We evaluated additional cases notified and conducted comparative interrupted time series (ITS) analyses to assess the impact of ACF by human resource model on TB case notifications.

**Results:**

We verbally screened 321,020 persons in the community, of whom 70,439 were eligible for testing and 1138 of them started TB treatment. ACF activities resulted in a + 15.9% [95% CI: + 15.0%, + 16.7%] rise in All Forms TB notifications in the intervention areas compared to control areas. The ITS analyses detected significant positive post-intervention trend differences in All Forms TB notification rates between the intervention and control areas (*p* = 0.001), as well as between the employee and volunteer human resource models (*p* = 0.021).

**Conclusions:**

Both salaried and volunteer CHW human resource models demonstrated additionality in case notifications compared to routine case finding by the government TB program**.** The salaried employee CHW model achieved a greater impact on notifications and should be prioritized for scale-up, given sufficient resources.

## Background

Globally, 1.6 million people die of tuberculosis (TB) annually and an estimated 10 million people develop TB disease each year [1]. Despite concerted efforts, incidence is currently declining at less than 2% per annum [2]. If this trend continues, TB elimination will not be achieved until the year 2182 [3]. As for all infectious diseases, early detection and cure of incident cases is crucial to decrease transmission and halt the epidemic. In endemic countries, National TB Control Programs (NTP) are responsible for providing TB care and prevention to the population. However, in routine NTP operations case finding entails receiving health-seeking patients with little effort on actively searching for new TB cases. As a consequence, there are 3 million annual incident TB cases globally whom these NTPs fail to reach. This “detection gap” sustains transmission and mortality [4]. One strategy to close the detection gap is active case finding (ACF) [5]. This has led to a global surge in ACF initiatives beyond routine household contact tracing [6, 7]. These initiatives range from facility-based systematic screening to community-based interventions among vulnerable populations [8, 9]. These initiatives have also shown that ACF can find more people with TB at an earlier stage of disease progression [10, 11]. However, better evidence on impact and cost-effectiveness is needed if they are to be sustained through national public health policies and budgets [12].

A prerequisite for ending TB entails the optimal engagement of communities. Community engagement has long been recognized as a priority in health care [13, 14] and the fight against TB [15, 16]. Previously defined as “paraprofessionals or lay individuals with an in-depth understanding of the community culture and language” that “have received standardized job-related training of a shorter duration than health professionals” with the primary goal of providing “culturally appropriate health services to the community,” [17] community health workers (CHW) comprise a critical component of community engagement [18]. When effectively engaged, CHWs can raise health system capacity through decentralization and task-shifting [19, 20]. CHW’s often also reduce access barriers for vulnerable populations, so that their services are considered more patient-centric than institutionally provided care [21]. As such, CHW’s comprise an ideal group to implement ACF for TB in their communities. However, there remains substantial heterogeneity in the CHW models applied and gaps in understanding regarding their optimal means of engagement [17, 22].

Under most applied models, CHW receive either a salary or an incentive-driven compensation. Oftentimes performance-based incentives (PBI) are additionally offered to reward high performers [23]. While salaried CHWs have been associated with increased motivation and reduced attrition [23], volunteer CHW models are often more attractive to authorities and implementers due to their lower fixed costs. Irrespective of payment model, a consistent recommendation is that financial compensation should be commensurate with occupational demands [18]. It has further been noted that CHWs are generally poor and dependent on the remuneration for their livelihood. For this reason, the World Health Organization (WHO) recommends to limit the dominance of PBIs as part of the overall compensation package [24]. However, it remains unclear whether a higher fixed payment is associated with improved performance and greater impact. The comparative effectiveness between salaried and volunteer CHWs was therefore identified as a key knowledge gap in need of further studies [25].

Between 2014 and 2016, a community-based ACF project named PROPER CARE was piloted in Go Vap district, Ho Chi Minh City (HCMC), Viet Nam. This project employed CHWs on a full-time basis with a commensurate salary for ACF and case management. Despite positive results [26], aspirations of other district health authorities to scale up this ACF initiative were stifled by the resource demands of these salaried CHWs. In response, we devised the IMPACT-TB study (Implementing proven community-based active TB case finding interventions). Its aim was to scale up the ACF activities piloted under the PROPER CARE project, to measure changes in TB case notifications resulting from these ACF activities, and to compare the relative changes in TB case notifications between ACF implemented by incentivized volunteers and salaried employees.

## Methods

### Study setting

**Fig. 1:**
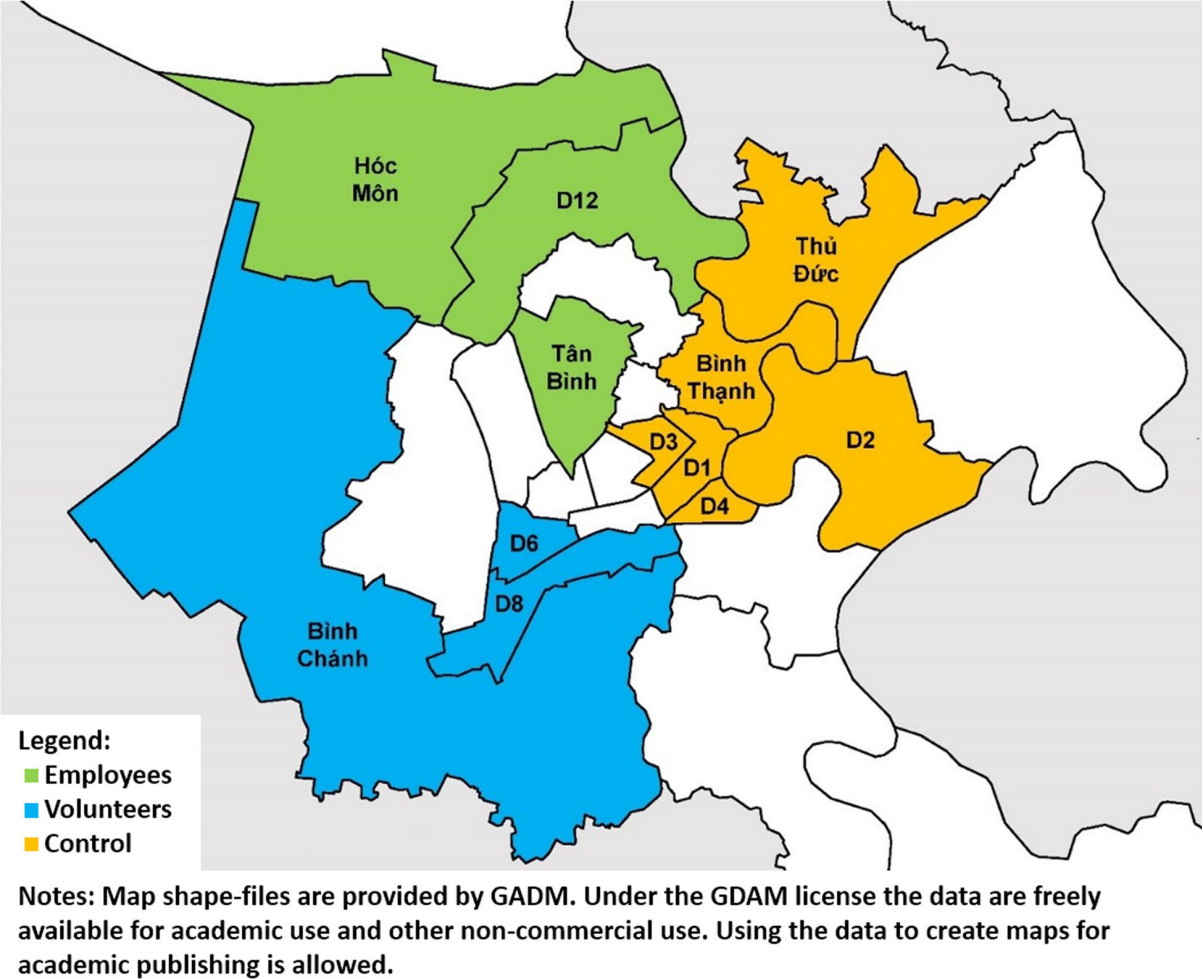
Location of intervention and control districts in Ho Chi Minh City, Viet Nam

The IMPACT-TB study was conducted in 12 of the 24 districts of HCMC (Fig. 1) between October 2017 and September 2019. Six intervention districts implemented ACF activities bifurcated by human resource model. In six control districts, routine TB care was provided by the NTP. The intervention districts were selected based on comparability of population size and TB burden, and absence of past or concurrent ACF activities. The human resource model was allocated in collaboration with the Pham Ngoc Thach Provincial TB Hospital (PNTH). The salaried employee model was implemented in Hoc Mon, Tan Binh and District 12. This area had a population of 1,465,819 and reported 1969 All Forms TB notifications in 2017. The volunteer model was implemented in Districts 6, 8 and Binh Chanh. This area had a population of 1,348,215 and reported 2190 All Forms TB notifications in 2017. The six control districts had a combined population of 1,789,396 and reported 2859 All Forms TB notifications in 2017. Each of the 12 project districts contained one District TB Unit (DTU) which received regular technical supervision from PNTH. The DTUs managed TB diagnosis, notification and treatment follow up in accordance with national guidelines.

### Community health workers

A cadre of CHWs was recruited in each intervention district to implement ACF activities. These individuals were identified by district authorities among current community volunteers, retired health staff, civil society members, and former TB patients. Residency in the district for over 5 years was a prerequisite to ensure geographic familiarity. CHWs were recruited by the district health authorities for the duration of the study as salaried employees or incentivized volunteers based on the study district. In our study, salaried employees were defined as full-time staff for the duration of the study, who focused solely on the ACF activities and TB patient support. These employees received a salary equivalent to USD 136/month. Volunteers had permission to engage in other livelihood generation activities. They were provided a stipend of USD 23/month to support study objectives. Both groups received the same training, core responsibilities and PBI schemes.

### Intervention

CHWs targeted household and close contacts of index cases, neighbors in proximity of an index case, and urban priority groups living in slum and boarding home settings (Supplemental Information, Figure S1). Index cases were prospective TB patients notified by the DTUs of the intervention districts. Household contacts were defined as people sharing a kitchen with an index case for one or more nights in the past 3 months. Close contacts were persons that interacted with an index patient at least once per month over the past 3 months. Proximity was defined as the same administrative neighborhood or in a 50 m catchment area around the residence of the index case [27]. We used United Nations Habitat definitions for slum households [28]. Boarding homes were defined as dormitories and single-room rental facilities. District authorities further helped to identify boarding home and urban poor communities for door-to-door screening.

Household contact investigations consisted of an enumeration of all contacts and verbal symptom screening of those who were present at the time of the contact investigation. Screening of close contacts was conducted either via phone or pre-arranged meeting. To screen index case neighbors and urban priority groups, CHWs conducted door-to-door visits. A verbal symptom screen was administered using a standardized questionnaire in a custom-built app installed on an Android tablet (Supplemental information, Data sources & processing). The questionnaire asked about the presence and duration of the following symptoms: cough, hemoptysis, chest pain, dyspnea, fever, night sweats, fatigue, weight loss, and history of TB.

Although all household contacts were first verbally screened for TB symptoms, they were all referred for additional chest X-ray (CXR) screening irrespective of their clinical presentation. All other persons were referred for CXR screening if they reported any one of the aforementioned symptoms. Persons with parenchymal abnormalities on CXR were tested on the Xpert MTB/RIF assay (Cepheid; Sunnyvale, CA, USA). Individuals who did not obtain a CXR or had a normal CXR but exhibited TB-related symptoms were tested using smear microscopy. Symptomatic persons with negative sputum test results were evaluated by the DTU and PNTH for clinical diagnosis in accordance to national treatment guidelines.

Persons accessing care received a transport stipend and fully subsidized CXR at the DTU or weekend community screening events. CHWs transported sputum samples for persons unable to reach the laboratories. TB patients with rifampicin-susceptible TB were linked to treatment at the DTU. This included CHWs assisting with enrollment formalities, including support to furnish proof of residency. Rifampicin-resistant TB patients were referred to PNTH for further evaluation and treatment. CHWs followed up patients before and after enrollment for counseling and psychosocial support.

### Study population

The study population included all household contacts, as well as close contacts and urban priority groups with clinical symptoms for whom CHWs completed a verbal symptom screen. Screened persons that declined to participate were referred, but their information was excluded from the analysis.

### Study outcomes

The primary outcome was the additionality in All Forms TB notifications in the intervention area. The secondary outcome was the difference in All Forms TB additionality between the two human resource models. We calculated additionality using the double-difference approach [29]. This approach calculates the additive effect of the pre−/post intervention difference in case notifications, plus the concurrent notification trend difference in a control area.

### Statistical analyses

We described the TB care cascade [30] bifurcated by human resource model. We presented the double-difference additionality for All Forms TB notifications and for TB patients with bacteriologic confirmation. To assess the validity of the double-difference additionality, we conducted comparative interrupted time series (ITS) analyses of aggregate monthly TB case notification rates in two iterations. The first iteration compared the intervention and control areas. The second iteration compared the two human resource models. The ITS analyses employed segmented methods (Supplemental Information, Figure S2) applied to marginal log-linear Poisson regression models using the generalized estimating equation (GEE) approach. We tested for serial autocorrelation using the Cumby-Huizinga test with a cutoff of *p* < 0.1 and specified the model based on the lowest quasi-likelihood information criterion values. Statistical analyses were performed on Stata version 13 (StataCorp; College Station, TX, USA). As a large proportion of the double-difference additionality derived from a decline in notifications the control area, we conducted a post-hoc comparative ITS analysis between the study’s six control districts and eight non-study districts where no ACF had been conducted in the past 3 years (from a total of 12 non-study districts in HCMC). Hypothesis tests were two-sided and point estimates included 95% confidence intervals.

### Ethical considerations

Ethical approvals were granted by the Pham Ngoc Thach Hospital Institutional Review Board and the Liverpool School of Tropical Medicine Research Ethics Committee. Study implementation was approved by the Ho Chi Minh City People’s Committee. We obtained written informed consent from all participants and anonymized all patient data prior to analysis.

## Results

### ACF outputs

**Fig. 2:**
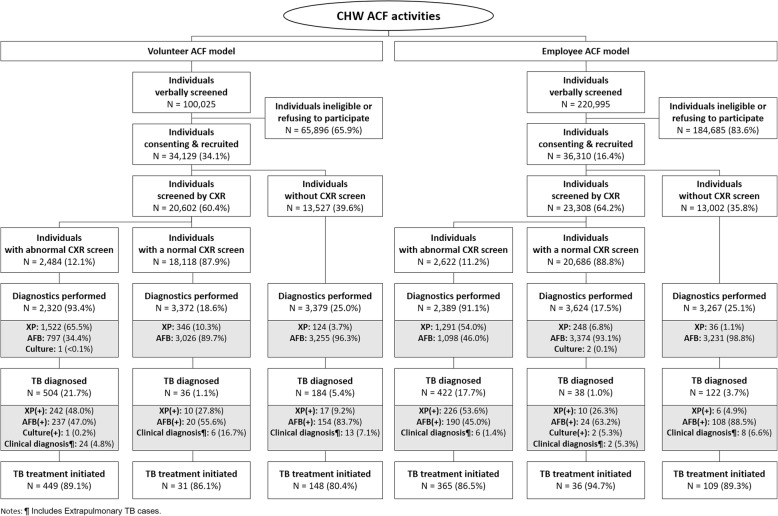
Process indicators disaggregated by human resource model and chest X-ray screening result

The TB care cascade is shown in Table 1 and Fig. 2. Over 2 years, CHWs verbally screened 321,020 people. Of those, 70,439 (21.9%) individuals were a household contact of a TB patient or had symptoms suggestive of TB and thus were eligible for further screening. A description of these individuals is in Table S1 of the supplemental information. We recorded CXR results for 62.3% (43,910/70,439) of these participants, among whom 11.6% (5106/43,910) had abnormalities. Xpert was used as the initial diagnostic test in 69.9% of persons with X-ray abnormalities (3567/5106). The Xpert positivity rate was 14.3% (511/3567). 14,781 people with no CXR result or a normal CXR were tested on smear microscopy with a positivity rate of 5.0% (733/14,781). Active TB was diagnosed in 0.4% (1306/321,020) of persons screened, of whom 87.1% (1138/1306) started NTP treatment. This represented a Number Needed to Screen (NNS) of 282 or a yield of 354 per 100,000.

**Table 1:** Process indicators disaggregated by human resource model

	Total N (%)	Volunteer ACF N (%)	Employee ACF N (%)
Individuals verbally screened	321,020 (100.0)	100,025 (100.0)	220,995 (100.0)
Individuals consenting & recruited^a^	70,439 (21.9)	34,129 (34.1)	36,310 (16.4)
Individuals eligible for CXR	59,781 (18.6)	29,438 (29.4)	30,343 (13.7)
Individuals screened by CXR	43,910 (13.7)	20,602 (20.6)	23,308 (10.5)
Individuals with abnormal CXR screen	5106 (1.6)	2484 (2.5)	2622 (1.2)
Individuals tested for TB (any sputum test)	18,351 (5.7)	9071 (9.1)	9280 (4.2)
Individuals tested for TB with Xpert	3567 (1.1)	1992 (2.0)	1575 (0.7)
Individuals tested for TB with Smear	14,781 (4.6)	7078 (7.1)	7703 (3.5)
Individuals tested for TB with Culture	3 (0.0)	1 (0.0)	2 (0.0)
Individuals diagnosed with All Forms TB	1306 (0.4)	724 (0.7)	582 (0.3)
Individuals diagnosed Xpert(+)	511 (0.2)	269 (0.3)	242 (0.1)
Individuals diagnosed Smear(+)	733 (0.2)	411 (0.4)	322 (0.1)
Individuals diagnosed Culture(+)	3 (< 0.1)	1 (< 0.1)	2 (< 0.1)
Individuals clinically diagnosed^b^	59 (< 0.1)	43 (< 0.1)	16 (< 0.1)
All Forms TB patients started on treatment	1138 (0.4)	628 (0.6)	510 (0.2)
*NNS / Yield*	*282 (0.4)*	*159 (0.6)*	*433 (0.2)*

^a^Comprised of household contacts and symptomatic persons from other target groups that consented to participate in the study;

^b^Includes extrapulmonary TB

While most indicators along the TB care cascade were similar between the human resource models, there were some notable differences. The number of individuals verbally screened by salaried employees (220,995) was 121% higher compared to volunteers (100,025). Conversely, the number of patients started on TB treatment in the salaried employee districts (510) was 19% lower than those started on treatment in the volunteer districts (628). As such, the NNS in for salaried employee districts (433) was 2.7 times higher than for volunteer districts (159).

### Notification impact

Changes in TB notifications for primary and secondary outcomes are shown in Table 2. The cumulative additionality in All Forms TB notifications in all intervention districts was + 15.9% [+ 15.0%, + 16.7%], corresponding to 1090 [1031, 1149] additional cases notified over 2 years. Pre−/post-implementation notification trend differences were + 5.0% [+ 4.5%, + 5.5%] in the intervention compared to − 10.9% [− 11.6%, − 10.1%] in the control districts. Bacteriologically-confirmed TB notifications rose + 22.0% [+ 20.8%, + 23.2%] corresponding to 1074 [1017, 1131] additional cases. Between pre- and post-implementation periods, notifications increased + 14.5% [+ 13.5%, + 15.4%] in the intervention area compared to a decline of − 7.5% [− 8.3%, − 6.7%] in the control.

**Table 2:** Additionality analysis [29] by study area and human resource model

	Cumulative notifications		Trend difference			
	Baseline period^a^	Intervention period	# cases	[95% CI]	% change^b^	[95% CI]
All forms TB						
Notification impact^d^			1090	[1031, 1149]	15.9%	[15.0%, 16.7%]
*By study area*						
Intervention area	8796	9236	440	[400, 480]	5.0%	[4.5%, 5.5%]
Control area	5988	5338	− 650	[− 697, − 603]	−10.9%	[− 11.6%, − 10.1%]
*By human resource model*						
Volunteer ACF			480	[439, 521]	8.8%	[8.0%, 9.6%]
Pre vs. post: Intervention	4580	4722	142	[119, 165]	3.1%	[2.6%, 3.6%]
Pre vs. post: Control^c^	3118	2779	−338	[−376, −306]	−5.7%	[−6.3%, −5.1%]
Employee ACF			610	[565, 655]	12.3%	[11.4%, 13.2%]
Pre vs. post: Intervention	4216	4514	298	[265, 331]	7.1%	[6.3%, 7.8%]
Pre vs. post: Control^c^	2870	2559	− 312	[− 345, − 278]	−5.2%	[−5.8%, −4.6%]
Bacteriologically-confirmed TB						
Notification impact^d^			1074	[1017, 1131]	22.0%	[20.8%, 23.2%]
*By study area*						
Intervention area	5402	6183	781	[730, 832]	14.5%	[13.5%, 15.4%]
Control area	3884	3591	− 293	[− 325, − 261]	−7.5%	[−8.4%, −6.7%]
*By human resource model*						
Volunteer ACF			401	[364, 438]	12.9%	[11.7%, 14.0%]
Pre vs. post: Intervention	2782	3032	250	[220, 280]	9.0%	[7.9%, 10.0%]
Pre vs. post: Control^c^	2000	1849	− 151	[− 175, −128]	−3.9%	[−4.5%, −3.3%]
Employee ACF			673	[629, 717]	23.9%	[22.3%, 25.5%]
Pre vs. post: Intervention	2620	3151	531	[491, 571]	20.3%	[18.7%, 21.8%]
Pre vs. post: Control^c^	1884	1742	− 142	[− 167, −121]	−3.7%	[−4.3%, − 3.1%]

^a^The baseline period consists of the October 2016–September 2017 timeframe; the cumulative baseline notifications are the sum of notifications matched by quarter to the intervention period of October 2017–September 2019;

^b^The sums of the percentage point estimates include rounding effects;

^c^The absolute and percent differences in pre vs. post intervention notifications in the control area were allocated to each human resource model based on their relative proportion of baseline notifications (Volunteer ACF: 4580/8796 = 52.1% vs Employee ACF: 4216/8796 = 47.9%);

^d^The number of cases denotes the double difference between pre- and post-implementation and between intervention and control areas;

The additionality in All Forms TB notifications disaggregated by human resource model was + 8.8% [+ 8.0%, + 9.6%] from volunteer districts compared to + 12.3% [+ 11.4%, + 13.2%] from salaried employee districts. These rates corresponded to 480 [439, 521] additional All Forms TB notifications in volunteer districts and 610 [565, 655] in salaried employee districts. With respect to bacteriologically-confirmed cases, volunteer districts raised notifications by an additional + 12.9% [+ 11.7%, + 14.0%] corresponding to 401 [364, 438] additional cases. The additionality in salaried employee districts was + 23.9% [+ 22.3%, + 25.5%] corresponding to 673 [629, 717] additional cases.

### Secondary and post-hoc analyses

**Fig. 3:**
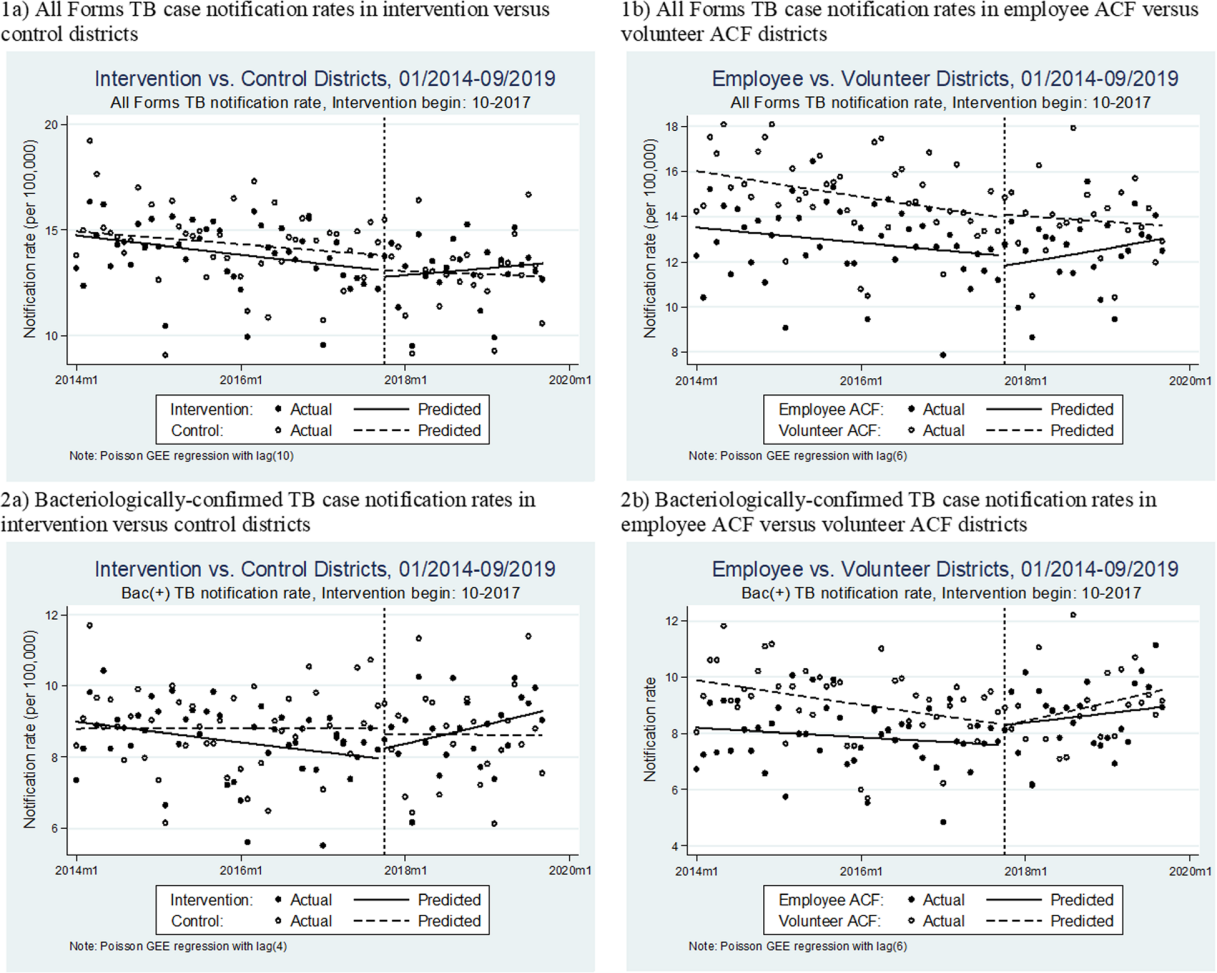
Comparative ITS analysis model graphs of population-standardized monthly notification rates of 1) All Forms TB case notification rates; and 2) bacteriologically-confirmed TB case notification rates for a) intervention versus control districts; and b) employee ACF versus volunteer ACF

The time series data consisted of 138 monthly aggregate counts of treatment notifications balanced between intervention and control districts. The monthly median All Forms TB notifications was 383 (Inter-quartile range: 358–403) in the intervention area and 249 (IQR: 233–265) in the control area. The ITS analyses results are in Table 3 and Fig. 3. In the post-implementation period, there was a significant trend difference between the intervention and control areas in All Forms TB (Incidence rate ratio(β_7_) = 1.004 [1.002, 1.006]; *p* = 0.001) and bacteriologically-confirmed TB notification rates (IRR(β_7_) = 1.008 [1.003, 1.014]; *p* = 0.002). Regarding the human resource models, the ITS analysis detected evidence of a post-intervention trend difference in favor of the salaried employee district over the volunteer districts in All Forms TB case notification rate (IRR(β_7_) = 1.005 [1.001, 1.009]; *p* = 0.021).

**Table 3:** Comparative ITS analysis model parameters^a^ of population-standardized monthly notification rates of All Forms and bacteriologically-confirmed TB cases for a) intervention versus control districts; and b) employee ACF versus volunteer ACF

	Intervention versus Control			Employee ACF versus Volunteer ACF		
	IRR^c^	95% CI	***p***-value^**d**^	IRR^c^	95% CI	***p***-value^**d**^
**All Forms TB**						
Baseline rate^b^ (*β*_*0*_)	14.931	[14.721, 15.144]	< 0.001	16.028	[15.643, 16.423]	< 0.001
Pre-intervention trend, control (*β*_*1*_)	0.998	[0.998, 0.999]	< 0.001	0.997	[0.996, 0.998]	< 0.001
Post-intervention step change, control (*β*_*2*_)	0.949	[0.921, 0.977]	< 0.001	1.011	[0.966, 1.059]	0.634
Post-intervention trend, control (*β*_*3*_)	1.001	[0.999, 1.003]	0.432	1.002	[0.999, 1.004]	0.264
Difference in baseline (*β*_*4*_)	0.987	[0.970, 1.006]	0.176	0.843	[0.814, 0.873]	< 0.001
Difference in pre-intervention trends (*β*_*5*_)	0.999	[0.998, 1.000]	0.014	1.001	[1.000, 1.002]	0.174
Difference in post-intervention step change (*β*_*6*_)	1.030	[0.992, 1.070]	0.123	0.953	[0.893, 1.018]	0.155
Difference in post-intervention trends (*β*_*7*_)	1.004	[1.002, 1.006]	0.001	1.005	[1.001, 1.009]	0.021
**Bacteriologically-confirmed TB**						
Baseline rate^b^ (*β*_*0*_)	8.793	[8.466, 9.133]	< 0.001	9.898	[9.559, 10.249]	< 0.001
Pre-intervention trend, control (*β*_*1*_)	1.000	[0.999, 1.002]	0.968	0.996	[0.995, 0.998]	< 0.001
Post-intervention step change, control (*β*_*2*_)	0.984	[0.920, 1.051]	0.628	0.996	[0.933, 1.064]	0.910
Post-intervention trend, control (*β*_*3*_)	1.000	[0.996, 1.004]	0.892	1.010	[1.006, 1.014]	< 0.001
Difference in baseline (*β*_*4*_)	1.023	[0.974, 1.074]	0.367	0.828	[0.787, 0.871]	< 0.001
Difference in pre-intervention trends (*β*_*5*_)	0.997	[0.995, 0.999]	0.005	1.002	[1.000, 1.004]	0.034
Difference in post-intervention step change (*β*_*6*_)	1.055	[0.969, 1.150]	0.218	1.098	[1.001, 1.204]	0.048
Difference in post-intervention trends (*β*_*7*_)	1.008	[1.003, 1.014]	0.002	0.995	[0.989, 1.000]	0.069

^a^The parameters were obtained for a segmented regression model with the following structure: *Y*_*t*_ = *β*_0_ + *β*_1_*T*_*t*_ + *β*_2_*X*_*t*_ + *β*_3_*X*_*t*_*T*_*t*_ + *β*_4_*Z* + *β*_5_*ZT*_*t*_ + *β*_6_*ZX*_*t*_ + *β*_6_*ZX*_*t*_*T*_*t*_ + *ϵ*_*t*_. Here Y_t_ is the outcome measure along time t; T_t_ is the monthly time counter; X_t_ indicates pre- and post-intervention periods, Z denotes the intervention cohort, and ZT_t_, ZX_t_, and ZX_t_T_t_ are interaction terms. β_0_ to β_3_ relate to the control group as follows: β_0_, intercept; β_1_, pre-intervention trend; β_2_, post-intervention step change; β_3_, post-intervention trend. β_4_ to β_7_ represent differences between the control and intervention districts: β_4_, difference in baseline intercepts; β_5_, difference in pre-intervention trends; β_6_, difference in post-intervention step changes; β_7_, difference in post-intervention trend

^b^The baseline rate denotes case notification rates per month

^c^IRR based on log-linear GEE Poisson regression with correlation structures determined by the Cumby-Huizinga test and Quasi-Information Criteria

^d^Wald test

The comparison between the study’s control districts and eight selected non-study districts showed no statistical difference in step-change (β_6_) or trends (β_7_) for bacteriologically-confirmed and All Forms TB notifications (Table 4).

**Table 4:** Comparative ITS analysis model parameters of population-standardized quarterly notification rates of All Forms and bacteriologically-confirmed TB cases for control versus non-IMPACT-TB districts^a,b,c^

	Control versus non-IMPACT-TB districts		
	IRR^d^	95% CI	***p***-value^**e**^
**All Forms TB**			
Baseline rate (*β*_*0*_)	48.104	[47.315, 48.906]	< 0.001
Pre-intervention trend, control (*β*_*1*_)	0.995	[0.994, 0.997]	< 0.001
Post-intervention step change, control (*β*_*2*_)	0.795	[0.763, 0.829]	< 0.001
Post-intervention trend, control (*β*_*3*_)	1.006	[0.997, 1.015]	0.168
Difference in baseline (*β*_*4*_)	0.802	[0.785, 0.821]	< 0.001
Difference in pre-intervention trends (*β*_*5*_)	1.006	[1.004, 1.008]	< 0.001
Difference in post-intervention step change (*β*_*6*_)	1.012	[0.957, 1.071]	0.667
Difference in post-intervention trends (*β*_*7*_)	1.006	[0.994, 1.018]	0.322
**Bacteriologically-confirmed TB**			
Baseline rate (*β*_*0*_)	27.461	[26.677, 28.268]	< 0.001
Pre-intervention trend, control (*β*_*1*_)	1.001	[0.998, 1.003]	0.599
Post-intervention step change, control (*β*_*2*_)	0.882	[0.825, 0.943]	< 0.001
Post-intervention trend, control (*β*_*3*_)	1.006	[0.992, 1.020]	0.372
Difference in baseline (*β*_*4*_)	0.810	[0.779, 0.843]	< 0.001
Difference in pre-intervention trends (*β*_*5*_)	1.003	[1.000, 1.006]	0.053
Difference in post-intervention step change (*β*_*6*_)	1.058	[0.968, 1.157]	0.217
Difference in post-intervention trends (*β*_*7*_)	0.994	[0.976, 1.013]	0.553

^a^The parameters were obtained for a segmented regression model with the following structure: *Y*_*t*_ = *β*_0_ + *β*_1_*T*_*t*_ + *β*_2_*X*_*t*_ + *β*_3_*X*_*t*_*T*_*t*_ + *β*_4_*Z* + *β*_5_*ZT*_*t*_ + *β*_6_*ZX*_*t*_ + *β*_6_*ZX*_*t*_*T*_*t*_ + *ϵ*_*t*_. Here Y_t_ is the outcome measure along time t; T_t_ is the monthly time counter; X_t_ indicates pre- and post-intervention periods, Z denotes the intervention cohort, and ZT_t_, ZX_t_, and ZX_t_T_t_ are interaction terms. β_0_ to β_3_ relate to the control group as follows: β_0_, intercept; β_1_, pre-intervention trend; β_2_, post-intervention step change; β_3_, post-intervention trend. β_4_ to β_7_ represent differences between the control and intervention districts: β_4_, difference in baseline intercepts; β_5_, difference in pre-intervention trends; β_6_, difference in post-intervention step changes; β_7_, difference in post-intervention trend

^b^The baseline rate denotes case notification rates per quarter as monthly notification rates were unavailable for non-study districts

^c^The non-IMPACT-TB districts included eight of the 12 remaining districts in HCMC. Four districts were excluded due to concurrent ACF interventions (Go Vap, 7 and 10) and large differences in population growth (Nha Be)

^d^IRR based on log-linear GEE Poisson regression with correlation structures as determined by the Cumby-Huizinga test and Quasi-Information Criteria

^e^Wald test

## Discussion

Our study demonstrated that community-based ACF can generate a substantial yield of previously undetected TB, even in a setting with a well-functioning TB program. Our ITS analyses substantiated the additionality calculations of the intervention by adjusting for changes in population size and seasonality. Although the trend differences in case notification rates were modest, they were statistically significant, supporting the case for a positive intervention impact. The post-hoc comparison between the study’s control and selected non-study districts further substantiated this finding by showing that the notification declines recorded in the study’s control areas were experienced across the majority of all non-study districts in HCMC.

Our results are concordant with those of community-based ACF studies conducted in other settings [31–36]. A key success factor of these studies was expanding screening coverage to large portions of vulnerable populations and enabling access to the more sensitive CXR-Xpert diagnostic algorithm. Another commonality was leveraging existing healthcare structures. Successful community engagement projects tended to be complementary to facility-based case finding [37]. The engagement of established networks has been shown to be effective in other health areas as well [38]. A final similarity entailed the strong community linkages. Our CHWs coordinated with neighborhood leaders and commune TB officers to conduct household contact investigations. This raised community confidence in the study and lowered access barriers [39].

In evaluating the two human resource models, we found both to be successful in improving TB detection and notification. Our analysis further showed that these yields did not displace routine activities in either model. This suggests that there is a general benefit to engaging CHWs for community-based TB service coverage expansion. We further found that despite a lower case detection yield, the increases in All Forms TB notifications in the salaried employee districts was significantly higher than the gains in the volunteer districts. This finding was substantiated by the ITS analysis.

One explanation for the dichotomy in yield and additionality was the difference in screening activities. It is well documented that to find more cases a greater number of people need to be screened [40]. The higher number of screening encounters by the employees can be a proxy for time spent on outreach. The ability of full-time CHWs to achieve higher population coverage compared to volunteers has been noted in other settings [41]. Greater population coverage and community outreach have been cited as a catalyst for increased notifications [42].

Reports from the field also suggest that volunteers placed greater reliance on public health staff and neighborhood leaders to refer persons with suspected TB. This is intuitive given the lower remuneration and time commitment to ACF activities. Consequently, a greater proportion of individuals initiated on the TB care pathway by volunteers would have potentially been notified without this ACF intervention. This displacement of passive case finding has been documented among household contacts in Viet Nam [43].

However, as evinced by the PROPER CARE pilot [26], there is limited utility in an effective community engagement model that government stakeholders deem untenable for scale-up. As such, another positive finding of our study is that the volunteer human resource model still resulted in a significant increase in TB notifications for a lower cost. However, if resource-constrained programs choose to implement a volunteer-based human resource model, it will be necessary to consider other inherent risks besides lower additionality that may offset anticipated cost savings. Volunteers tend to require greater supervisory efforts to improve performance and ensure accurate reporting [25, 44]. Past studies have also found volunteerism to be associated with lower value perception and job satisfaction [45]. Concordantly, the attrition in volunteer districts was higher than in salaried employee districts. This was also the case among district supervisors. The attrition required continuous recruitment and capacity building, and possibly impaired quality of care. These downsides of volunteerism have been noted elsewhere [46, 47]. A health economic analysis that incorporates the cost implications of the attrition and other operational challenges encountered in our study will be provided in a separate manuscript.

The Viet Nam NTP has already successfully replicated the volunteer model in cities with a lower TB burden and cost of living compared to HCMC, the economic center of Viet Nam [48]. In these cities the model generated comparable notification increases to the employee model in HCMC (manuscript in preparation). This suggests that the volunteer model could be appropriate in settings, where the workload and the opportunity cost of volunteering are lower. Appropriately powering incentives for the socioeconomic context and employing a blend of monetary and non-monetary incentives are further means to optimize volunteer models [49]. Lastly, future research may seek better ways to draw on the altruistic capital of volunteer CHWs to overcome operational challenges [50].

Our study had limitations. Conducting research in a programmatic setting exposed our study to supply chain interruptions. These interruptions affected the integration of study activities into routine program operations. However, this also reflects the reality of field scale-up of an intervention. While ITS analyses aim to increase validity and interpretability of the results by compensating for secular trends, we may have missed nuanced confounders due to the non-randomized study design. A strength of our study was the length and scale of our outreach as well as integration within the existing NTP activities. As such, we believe in the transferability of our findings that effective engagement of CHWs can produce a positive notification impact and that full-time employment of CHWs can generate superior outcomes to other high TB burden, resource-limited settings to inform local strategies to end TB.

## Conclusions

Leveraging community networks to expand TB service coverage is both feasible and effective in diagnosing and treating additional persons with TB. Engaging full-time, salaried employee CHWs in TB ACF schemes can lead to greater impact, and this human resource model should be prioritized for scale up where resources permit. While further studies are needed to optimize this community engagement model, it can be a powerful and readily available tool for advancing the global End TB Strategy targets.

## Supplementary information

**Additional file 1: Figure S1.** Active TB case finding algorithm. **Figure S2.** Visualization of the comparative interrupted time-series analysis (intervention = upper line, control = lower line). **Table S1.** Demographic and clinical characteristics of study participants.

## Data Availability

The data that support the findings of this study are available from the Viet Nam National TB Control Program and Pham Ngoc Thach Provincial TB Hospital, but restrictions apply to the availability of these data. Data are can be made available from the authors upon reasonable request and with permission of the Viet Nam National TB Control Program and Pham Ngoc Thach Provincial TB Hospital.

## Abbreviations

ACF: Active Case Finding
AFB: Acid-fast Bacilli
CHW: Community Health Worker
CI: Confidence Interval
CXR: Chest X-ray
DTU: District TB Unit
EP: Extra-pulmonary
GEE: Generalized Estimating Equation
HCMC: Ho Chi Minh City
IRR: Incidence Rate Ratio
ITS: Interrupted Time Series
NNS: Number Needed to Screen
NTP: National TB Control Program
PBI: Performance-based Incentive
PNTH: Pham Ngoc Thach Provincial TB Hospital
TB: Tuberculosis
WHO: World Health Organization
