# Supplementary material for: A comparative impact evaluation of two human resource models for community-based active tuberculosis case finding in Ho Chi Minh City, Viet Nam
Source: BMC Public Health. 2020 Jun 15;20:934. doi: 10.1186/s12889-020-09042-4 (PMC7296629; doi:10.1186/s12889-020-09042-4)
Supplement: Supplementary file 1 — Additional file 1: Figure S1. Active TB case finding algorithm. Figure S2. Visualization of the comparative interrupted time-series analysis (intervention = upper line, control = lower line). Table S1. Demographic and clinical characteristics of study participants. [file 12889_2020_9042_MOESM1_ESM.docx]

# Supplemental information

## Supplementary methods

Figure S1: Active TB case finding algorithm


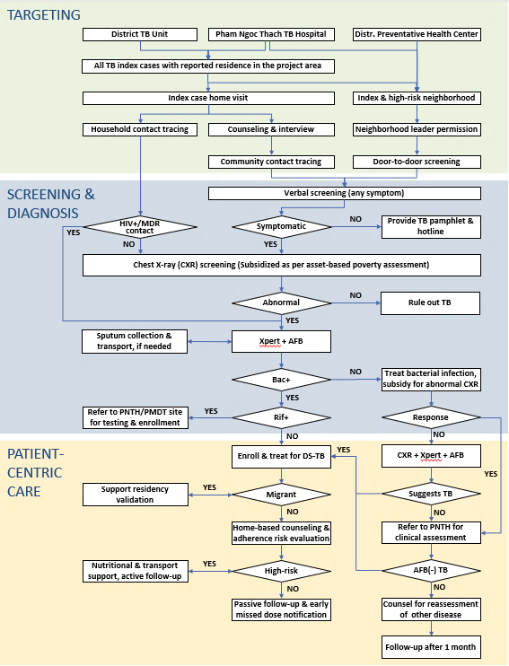


## Data sources & processing

To collect study data we used a bespoke mHealth application with connected web-platform titled ACIS (Access to Care Information System, Clinton Health Access Initiative/TechUp, Viet Nam). This app was a simple survey tool that collected responses and stored them in basic relational databases. The app’s unique feature was its bidirectional API that connected the app with the National TB Programme’s electronic recording and reporting system, VITIMES. This connectivity eliminated the necessity for field staff to request personal identifying information during household visits and reduced redundancy in the data collection process. A codebook and templates of all data collection forms were created to standardize data capture. Data validations were included as part of the app design. The ACIS system and these validations were pre-tested in a laboratory environment and piloted in the field. All data collected on ACIS were stored in the cloud and on physical servers in Viet Nam with redundancy in Taiwan and inaccessible by field staff after completion of the data collection process, so that patient confidentiality was preserved in the end of loss or theft of a tablet. Outside of the ACIS environment, all data were stored and managed via HIPAA (Health Insurance Portability and Accountability Act) compliant cloud storage. Local data was stored on study-specific, password-protected computers. Physical documents and audio records were stored in lockable cabinets in the FIT office. CHWs were trained in the use of an Android tablet and the data collection procedures via the ACIS app. CHWs used the ACIS mHealth app to record household contact investigations, and symptomatic screening encounters. The total number of symptomatic and asymptomatic screening encounters was reported as a monthly aggregate. A 7-digit unique ACIS ID number was auto-generated for each individual record created in ACIS. This ACIS ID was used for subsequent matching and data collection. District-level site coordinators reviewed screening encounter data and added CXR, diagnostic and treatment linkage results. These data were obtained from routine surveillance records and entered via the ACIS web-platform. A data quality team reviewed and processed these field data. Data processing included removal of missing values, de-duplication and overall data quality assurance.

For the additionality and ITS analyses, we collected and analyzed retrospective clinical patient records from the NTP and all relevant clinical data collected within NTP’s routine surveillance system. Official NTP patient data were periodically reviewed and validated for quality and alignment with national and WHO case definitions by the NTP’s IT and surveillance team. Clinical and patient data were encrypted, transferred and stored in servers hosted by the NTP. As patient data were transferred directly from VITIMES to ACIS, all data were concordant with the official records of the NTP. However, study personnel could correct pre-loaded information, e.g., address, phone number, etc., if the information contained in VITIMES did not reflect the current reality. In this case, the research staff corrected the new information. However, the system did not overwrite any new information collected through ACIS. Instead, it had a built-in parallel structure, where VITIMES and ACIS information for each variable were stored side-by-side. This enabled both the preservation of the NTP records and the flexibility to alter the information in non-destructive fashion. Subsequent to data collection, two designated members of the study team reviewed all data collected via the ACIS web-portal to ensure fidelity and quality of the collected information. Patient data received through the API were triangulated using district-level paper registers and aggregate reports from the NTP.

## Data analysis

$$Y_{t}= \beta_{0}+\beta_{1}T_{t}+\beta_{2}X_{t}+\beta_{3}X_{t}T_{t}+\beta_{4}Z+\beta_{5}ZT_{t}+\beta_{6}ZX_{t}+\beta_{6}ZX_{t}T_{t}+\epsilon_{t}$$

Figure S2 shows a visualization of the segmented regression model. The model measures differences in post-intervention step-changes and trends. Here Y_t_ is the outcome measure along time t; T_t_ is the monthly time counter; X_t_ indicates pre- and post-intervention periods, Z denotes the intervention cohort, and ZT_t,_ ZX_t_, and ZX_t_T_t_ are interaction terms. β_0_ to β_3_ relate to the control group as follows: β_0_, intercept; β_1_, pre-intervention slope; β_2_, post-intervention step change; β_3_, post-intervention trend. β_4_ to β_7_ represent differences between the control and intervention districts: β_4_, difference in baseline intercepts; β_5_, difference in pre-intervention trends; β_6_, difference in post-intervention step changes; β_7_, post-intervention trend difference.

Figure S2: Visualization of the comparative interrupted time-series analysis (intervention = upper line, control = lower line)


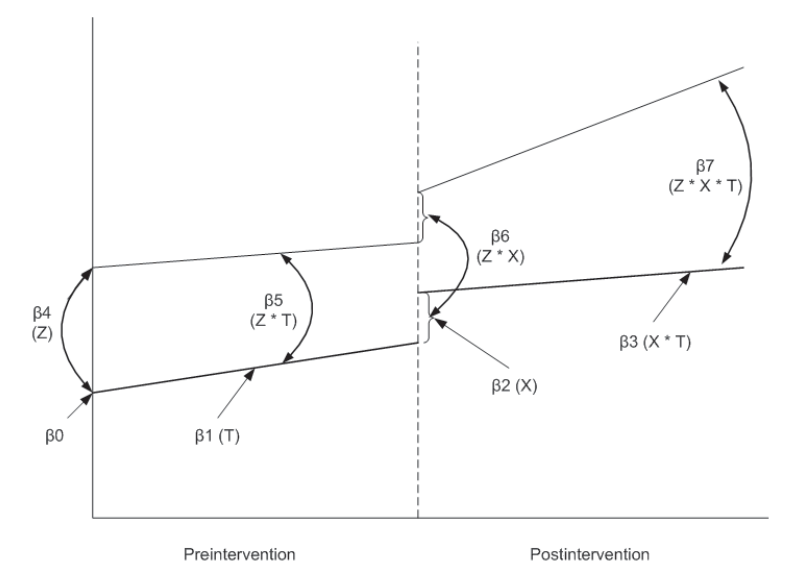


# Supplementary results

**Table S1: Demographic and clinical characteristics of study participants**

|  | **Total**  **(N = 70,439)**  **N (%)** | **Volunteer ACF**  **(N = 34,129)**  **N (%)** | **Employee ACF**  **(N = 36,310)**  **N (%)** |
| --- | --- | --- | --- |
| **Sex**¶ |  |  |  |
| Male | 31,968 / 70,212 (45.5) | 15,736 / 34,034 (46.2) | 16,232 / 36,178 (44.9) |
| Female | 38,244 / 70,212 (54.5) | 18,298 / 34,034 (53.8) | 19,946 / 36,178 (55.1) |
| **Age**¶ |  |  |  |
| <15 years | 4,526 / 68,986 (6.6) | 1,931 / 33,168 (5.8) | 2,595 / 35,818 (7.2) |
| 15-29 years | 9,543 / 68,986 (13.8) | 4,588 / 33,168 (13.8) | 4,955 / 35,818 (13.8) |
| 30-44 years | 14,717 / 68,986 (21.3) | 7,083 / 33,168 (21.4) | 7,634 / 35,818 (21.3) |
| 45-59 years | 19,688 / 68,986 (28.5) | 9,483 / 33,168 (28.6) | 10,205 / 35,818 (28.5) |
| >60 years | 20,512 / 68,986 (29.7) | 10,083 / 33,168 (30.4) | 10,429 / 35,818 (29.1) |
| **Urbanization** |  |  |  |
| Peri-urban | 25,854 (36.7) | 15,481 (45.4) | 10,373 (28.6) |
| Urban | 44,585 (63.3) | 18,648 (54.6) | 25,937 (71.4) |
| **Target group**¶ |  |  |  |
| Household contacts | 23,693 (33.6) | 12,266 (35.9) | 11,427 (31.5) |
| Close contacts | 2,977 (4.2) | 906 (2.7) | 2,071 (5.7) |
| Vulnerable populations | 43,769 (62.1) | 20,957 (61.4) | 22,812 (62.8) |
| **Social health insurance**¶ |  |  |  |
| No | 11,046 / 70,342 (15.7) | 5,516 / 34,067 (16.2) | 5,530 / 36,275 (15.2) |
| Yes | 59,296 / 70,342 (84.3) | 28,551 / 34,067 (83.8) | 30,745 / 36,275 (84.8) |
| **Any TB symptoms**┼ |  |  |  |
| No | 26,406 (37.5) | 14,210 (41.6) | 12,196 (33.6) |
| Yes | 44,033 (62.5) | 19,919 (58.4) | 24,114 (66.4) |
| **Four main TB symptoms**§ |  |  |  |
| No | 32,406 (46.0) | 17,693 (51.8) | 14,713 (40.5) |
| Yes | 38,033 (54.0) | 16,436 (48.2) | 21,597 (59.5) |
| **Cough any duration** |  |  |  |
| No | 35,691 (50.7) | 18,790 (55.1) | 16,901 (46.6) |
| Yes | 34,748 (49.3) | 15,339 (44.9) | 19,409 (53.5) |
| **Cough 2 weeks** |  |  |  |
| No | 47,842 (67.9) | 23,811 (69.8) | 24,031 (66.2) |
| Yes | 22,597 (32.1) | 10,318 (30.2) | 12,279 (33.8) |
| **Previous history of TB** |  |  |  |
| No/Unknown | 66,362 (94.2) | 31,858 (93.4) | 34,504 (95.0) |
| Yes | 4,077 (5.8) | 2,271 (6.7) | 1,806 (5.0) |
| **Chest X-ray result** |  |  |  |
| No CXR | 26,529 (37.7) | 13,527 (39.6) | 13,002 (35.8) |
| Normal | 38,804 (55.1) | 18,118 (53.1) | 20,686 (57.0) |
| Abnormal | 5,106 (7.3) | 2,484 (7.3) | 2,622 (7.2) |
| **Primary bacteriologic test** |  |  |  |
| No bacteriologic test | 52,088 (74.0) | 25,058 (73.4) | 27,030 (74.4) |
| Sputum smear microscopy | 14,781 (21.0) | 7,078 (20.7) | 7,703 (21.2) |
| Xpert MTB/RIF | 3,567 (5.1) | 1,992 (5.8) | 1,575 (4.3) |
| Culture | 3 (0.0) | 1 (0.0) | 2 (0.0) |
| **TB diagnosis** |  |  |  |
| No TB | 69,133 (98.2) | 33,405 (97.9) | 35,728 (98.4) |
| Active TB | 1,306 (1.9) | 724 (2.1) | 582 (1.6) |
| Bacteriologic confirmation | 1,247 (1.8) | 681 (2.0) | 566 (1.6) |
| Clinical diagnosis | 59 (0.1) | 43 (0.1) | 16 (0.0) |
| **Linkage to care**¶ |  |  |  |
| Initial loss to follow-up | 168 (0.2) | 96 (0.3) | 72 (0.2) |
| Enrolled onto treatment | 1,138 (1.6) | 628 (1.8) | 510 (1.4) |

Notes:

¶ N sizes listed due to missing values or exclusion of not applicable results;

┼ Includes (productive) cough of any duration, hemoptysis, chest pain, fever, night sweats, and fatigue;

§ Includes cough of any duration, fever, night sweats and weight loss.
